# Growth inhibition and ultrastructural alterations induced by Δ^24(25)^-sterol methyltransferase inhibitors in *Candida *spp. isolates, including non-*albicans *organisms

**DOI:** 10.1186/1471-2180-9-74

**Published:** 2009-04-20

**Authors:** Kelly Ishida, Juliany Cola Fernandes Rodrigues, Marcos Dornelas Ribeiro, Taíssa Vieira Machado Vila, Wanderley de Souza, Julio A Urbina, Celso Vataru Nakamura, Sonia Rozental

**Affiliations:** 1 Laboratório de Biologia Celular de Fungos, Instituto de Biofísica Carlos Chagas Filho, Universidade Federal do Rio de Janeiro, Rio de Janeiro/RJ, Brazil; 2 Laboratório de Ultraestrutura Celular Hertha Meyer, Instituto de Biofísica Carlos Chagas Filho, Universidade Federal do Rio de Janeiro, Rio de Janeiro/RJ, Brazil; 3 Instituto de Biologia do Exército – IBEX, Rio de Janeiro, Brazil; 4 Instituto Estadual de Hematologia – Hemorio, Rio de Janeiro, Brazil; 5 Laboratorio de Química Biológica, Instituto Venezolano de Investigaciones Científicas, Caracas 1020, Venezuela; 6 Laboratório de Microbiologia Aplicada a Produtos Naturais e Sintéticos, Universidade Estadual de Maringá/PR, Brazil

## Abstract

**Background:**

Although *Candida *species are commensal microorganisms, they can cause many invasive fungal infections. In addition, antifungal resistance can contribute to failure of treatment.

The purpose of this study was to evaluate the antifungal activity of inhibitors of Δ^24(25)^-sterol methyltransferase (24-SMTI), 20-piperidin-2-yl-5α-pregnan-3β-20(R)-diol (AZA), and 24(R,S),25-epiminolanosterol (EIL), against clinical isolates of *Candida *spp., analysing the ultrastructural changes.

**Results:**

AZA and EIL were found to be potent growth inhibitors of *Candida *spp. isolates. The median MIC_50 _was 0.5 μg.ml^-1 ^for AZA and 2 μg.ml^-1 ^for EIL, and the MIC_90 _was 2 μg.ml^-1 ^for both compounds. All strains used in this study were susceptible to amphotericin B; however, some isolates were fluconazole- and itraconazole-resistant. Most of the azole-resistant isolates were *Candida *non-*albicans *(CNA) species, but several of them, such as *C. guilliermondii, C. zeylanoides*, and *C. lipolytica*, were susceptible to 24-SMTI, indicating a lack of cross-resistance. Reference strain *C. krusei *(ATCC 6258, FLC-resistant) was consistently susceptible to AZA, although not to EIL. The fungicidal activity of 24-SMTI was particularly high against CNA isolates. Treatment with sub-inhibitory concentrations of AZA and EIL induced several ultrastructural alterations, including changes in the cell-wall shape and thickness, a pronounced disconnection between the cell wall and cytoplasm with an electron-lucent zone between them, mitochondrial swelling, and the presence of electron-dense vacuoles. Fluorescence microscopy analyses indicated an accumulation of lipid bodies and alterations in the cell cycle of the yeasts. The selectivity of 24-SMTI for fungal cells versus mammalian cells was assessed by the sulforhodamine B viability assay.

**Conclusion:**

Taken together, these results suggest that inhibition of 24-SMT may be a novel approach to control *Candida *spp. infections, including those caused by azole-resistant strains.

## Background

*Candida *species are commensal microorganisms of vertebrate hosts that can cause infections ranging from non-life-threatening to invasive illnesses. Although candidaemia is the most common manifestation of invasive candidiasis, extensive visceral invasion with *Candida *can occur in all organs. The eyes, brain, liver, spleen, and kidneys are the most commonly affected [1]. Candidiasis is the fourth most common cause of nosocomial bloodstream infections in Brazil and the U.S.A., with a mortality rate of approximately 40% [1,2]. A progressive increase in the number and severity of candidiasis over the past two decades has been observed worldwide, especially in immunocompromised patients and also in patients hospitalised with serious underlying diseases, during immunosuppressive therapy, or parenteral nutrition, as well as among patients exposed to invasive medical procedures. Yeasts of *Candida albicans *are the most frequently implicated in cases of invasive candidiasis infections. However, nowadays *Candida *non-*albicans *(CNA) species such as *Candida glabrata*, *Candida krusei*, and *Candida parapsilosis *have increased in importance and number among fungal infections [1].

Currently, the mainstay of chemotherapy employed for the treatment of fungal infections comprises drugs that affect the function or biosynthesis of membrane sterols [3]. The polyenes (such as amphotericin B) were the first antifungal class used to treat invasive fungal infections. The primary mechanism of amphotericin B is its binding to the signature 24-alkyl sterols present in fungal cell membranes, leading to a perturbation of the membrane selective permeability and, consequently, loss of the cellular content. Despite the specific fungicidal effect of polyenes, they display significant toxicity to mammalian cells [4]. Another important antifungal class comprises the azoles, such as ketoconazole, fluconazole (FLC), itraconazole (ITC), posaconazole, and voriconazole, which are the compounds most frequently used today, and whose specific target is the cytochrome P-450-dependent C14α-demethylase, a key enzyme of the ergosterol biosynthesis pathway [4]. Although azoles are one of the main classes of drugs used in the treatment of fungal infections, these drugs present several problems such as their fungistatic rather than fungicidal activity, variable drug bioavailability, lack of intravenous preparations, large number of drug-drug interactions, development of resistance, and potential cross-resistance between different azoles [5].

**Figure 1:**
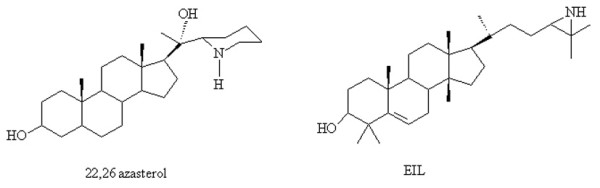
**Molecular structures of 20-piperidin-2-yl-5α-pregnan-3β,20-diol (22,26-azasterol, AZA) and 24 (R,S),25-epiminolanosterol (EIL)**.

During the last two decades, some studies have described a new class of antifungals called azasterols, which are inhibitors of the Δ^24(25)^-sterol methyltransferase (24-SMT), another key enzyme of the ergosterol biosynthesis pathway, which is absent in the mammalian host cells [6-8]. This enzyme catalyses the S-adenosylmethionine-mediated incorporation of methyl groups at position 24 in sterols, which is an essential step for the biosynthesis of fungal sterols [6,8]. 20-piperidin-2-yl-5α-pregnan-3β-20(R)-diol (AZA) and 24(R,S),25-epiminolanosterol (EIL) are steroid compounds with a nitrogen atom in the side chain (azasterols, Fig. 1), and are known inhibitors of 24-SMT in fungi [9], *Trypanosoma cruzi *[10], and *Leishmania amazonensis *[11,12]. Antifungal activities of these inhibitors were also described against *Pneumocytis carinii *[13] and *Paracoccidioides brasiliensis *[14].

The purpose of the present study was to (i) examine the susceptibilities of a collection of 70 yeasts of the genus *Candida *to AZA and EIL; (ii) determine the fungicidal activities of these compounds; and (iii) detect the main morphology and ultrastructural alterations of the yeasts after drug treatment.

## Results

### Antifungal susceptibility of Candida isolates

The MICs obtained for the ATCC strains to standard drugs (AMB, FLC, and ITC) and to the experimental compounds (AZA and EIL) are listed in Table 1. Interestingly, C. *krusei *(ATCC 6258, FLC-resistant) has AZA MIC_50 _of 1 μg.ml^-1 ^and MIC_90 _of 2 μg.ml^-1^. On the other hand, EIL did not inhibit the growth of the FLC- and ITC-resistant strains. All clinical isolates were susceptible to AMB, with the median MIC_50 _values ranging from 0.015 to 0.25 μg.ml^-1 ^and the MIC_90 _from 0.12 to 0.5 μg.ml^-1 ^(Table 2). However, three isolates (two *C. tropicalis *and one C. *guilhermondii*) showed MIC_90 _values higher than 1 μg.ml^-1^. Susceptibility to FLC was observed in 92% of the isolates, although 26% showed a trailing effect. Clear resistance to FLC was detected in three isolates (two *C. tropicalis *and one *C. krusei*). 45% of the strains showed MIC_50 _of 0.25–0.50 μg.ml^-1 ^and 37% showed MIC_90 _of 0.50–1 μg.ml^-1^. On the other hand, 75% of the isolates were susceptible to ITC, and 16% showed a trailing effect. Resistance to ITC was detected in 6 isolates (3 *C. tropicalis*, 1 *C. albicans*, 1 *C. glabrata*, and 1 *C. krusei*). Most of the isolates had MIC_50 _and MIC_90 _for ITC lower than 0.03 μg.ml^-1 ^(62%, and 41%, respectively). Only *C. krusei *isolates were less susceptible to all standard drugs, showing a MIC_90 _of 0.5 μg.ml^-1 ^for AMB, > 128 μg.ml^-1 ^for FLC, and 2 μg.ml^-1 ^for ITC (Table 2).

**Table 1:** Susceptibility of ATCC strains to Δ^24(25) ^sterol methyl transferase inhibitors, 20-piperidin-2-yl-5α-pregnan-3β, 20-diol (AZA) and 24 (R,S), 25-epiminolanosterol (EIL), and standard antifungals (FLC, ITC, and AMB) by the broth microdilution method.

Strains	AZA		EIL		FLC		ITC		AMB	
	MIC_50_	MIC_90_	MIC_50_	MIC_90_	MIC_50_	MIC_90_	MIC_50_	MIC_90_	MIC_50_	MIC_90_

*C. albicans *ATCC 10231	> 16	> 16	1	> 16	1	> 128^T^	0.5	> 16^T^	0.12	0.25
*C. parapsilosis *ATCC 22019	0.25	4	2	4	2	4	0.03	0.06	0.03	0.06
*C. tropicalis *ATCC 13803	0.25	4	1	2	0.25	2	< 0.03	0.03	0.007	0.25
*C. krusei *ATCC 6258	0.05	1	> 16	> 16	32	64^R^	0.12	0.25	0.25	0.25
*C. glabrata *ATCC 2001	1	2	> 16	> 16	4	> 128^T^	0.12	4^T^	0.03	0.12

^T^Trailing Effect, ^R^Resistant

The values are expressed in μg.ml^-1^.

**Table 2:** Antifungal susceptibilities of 65 clinical isolates of *Candida *spp. to amphotericin B (AMB), fluconazole (FLC), and itraconazole (ITC) by the CLSI reference broth microdilution method.

Antifungal	Species (no. of isolates)	Concentration (μg.ml^-1^)			Susceptibility no. isolates (%)		
		
		range of the MICs	^+^MIC_50_	^+^MIC_90_	S	SDD	R
AMB	All species (65)	≤ 0.007 – 1	0.06	0.12	65 (100)	-	
	*Candida albicans *(21)	≤ 0.007 – 0.5	0.06	0.12	21 (100)	-	
	*Candida parapsilosis *(19)	0.015 – 0.5	0.03	0.12	19 (100)	-	
	*Candida tropicalis *(14)	0.015 – 1	0.06	0.25	14 (100)	-	
	*Candida glabrata *(2)	0.015–0.5	0.12	0.25	2 (100)	-	
	*Candida krusei *(1)	0.25 – 0.5	0.25	0.5	1 (100)	-	
	*Candida lusitaneae *(1)	0.06 – 0.12	0.06	0.12	1 (100)	-	
	*Candida guilliermondii *(3)	0.015 – 1	0.015	0.06	3 (100)	-	
	*Candida zeylanoides *(1)	0.06 – 0.12	0.06	0.12	1 (100)	-	
	*Candida rugosa *(1)	0.03 – 0.12	0.03	0.12	1 (100)	-	
	*Candida dubliniensis *(1)	0.12 – 0.25	0.12	0.25	1 (100)	-	
	*Candida lipolytica *(1)	0.12 – 0.25	0.12	0.25	1 (100)	-	
							
FLC	All species (65)	≤ 0.25 – > 128*	0.5	1	60 (92.31)	2 (3.07)	3 (4.62)
	*Candida albicans *(21)	≤ 0.25 – > 128*	0.25	4	21 (100)		
	*Candida parapsilosis *(19)	≤ 0.25 – > 128*	0.5	0.5	19 (100)		
	*Candida tropicalis *(14)	≤ 0.25 – > 128*	0.5	4.5	12 (85.71)		2 (14.29)
	*Candida glabrata *(2)	≤ 0.25 – > 128*	4	64	2 (100)		
	*Candida krusei *(1)	16 – > 128	16	> 128			1 (100)
	*Candida lusitaneae *(1)	0.5 – 1	0.5	1	1 (100)		
	*Candida guilliermondii *(3)	0.12 – 16	4	4	2 (66.67)	1 (33.33)	
	*Candida zeylanoides *(1)	4 – 16	4	16		1 (100)	
	*Candida rugosa *(1)	0.5	0.5	0.5	1 (100)		
	*Candida dubliniensis *(1)	≤ 0.25 – 0.5	≤ 0.25	0.5	1 (100)		
	*Candida lipolytica *(1)	0.5 – 1	0.5	1	1 (100)		
							
ITC	All species (65)	≤ 0.03 – > 16**	≤ 0.03	0.12	49 (75.38)	10 (15.38)	6 (9.23)
	*Candida albicans *(21)	≤ 0.03 – > 16**	≤ 0.03	≤ 0.03	17 (80.95)	3 (14.28)	1 (4.76)
	*Candida parapsilosis *(19)	≤ 0.03 – > 16**	≤ 0.03	≤ 0.03	18 (94.74)	1 (5.26)	
	*Candida tropicalis *(14)	≤ 0.03 – > 16**	≤ 0.03	1.25	9 (64.28)	2 (14.28)	3 (21.43)
	*Candida glabrata *(2)	≤ 0.03 – 4	0.5	2		1 (50)	1 (50)
	*Candida krusei *(1)	0.12 – 2	0.5	2			1 (100)
	*Candida lusitaneae *(1)	≤ 0.03 – 0.12	≤ 0.03	0.12	1 (100)		
	*Candida guilliermondii *(3)	0.06 – 0.5	0.12	0.25	1 (33.33)	2 (66.66)	
	*Candida zeylanoides *(1)	0.06 – 0.12	0.06	0.12	1 (100)		
	*Candida rugosa *(1)	≤ 0.03	≤ 0.03	≤ 0.03	1 (100)		
	*Candida dubliniensis *(1)	0.06 – 0.12	0.06	0.12	1 (100)		
	*Candida lipolytica *(1)	0.25 – 0.5	0.25	0.5		1 (100)	

-Not determinate; ^+^MIC results are medians; *Trailing effect to FLC [*C. albicans *(9), *C. tropicalis *(4), *C. parapsilosis *(3) and one *C. glabrata*(1)]; **Trailing effect to ITC [*C. albicans *(6), *C. tropicalis *(4) and *C. parapsilosis *(1)].

When the MIC values for 24-SMTI (AZA and EIL) were analysed, we observed important antifungal activity for almost all *Candida *spp. isolates (Table 3). For AZA, MIC_50 _and MIC_90 _values lower than 2 μg.ml^-1 ^were observed in 86% and 52% of the isolates, respectively. For EIL, MIC_50 _and MIC_90 _values lower than 2 μg.ml^-1 ^were observed in 92% and 63% of the isolates, respectively. *C. zeylanoides *and *C. lipolytica *(a rarely observed CNA) showed MIC_50–90 _values of ≤ 0.03 μg.ml^-1 ^for both inhibitors, whilst *C. krusei *was resistant to EIL, with MIC_50–90 _values of 8 μg.ml^-1 ^(Table 3). However, both *C. krusei *and *C. lipolytica *were resistant to AZA (MIC_50–90 _≥ 16 μg.ml^-1^) (Table 3). Finally, *C. guilliermondii *isolates, FLC- and ITC-resistant, were susceptible to AZA, with MIC_50–90 _values of 0.06 – 0.25 μg.ml^-1^.

**Table 3:** Antifungal activity of 20-piperidin-2-yl-5α-pregnan-3β,20-diol (AZA) and 24 (R,S),25-epiminolanosterol (EIL), Δ^24(25)^-sterol methyl transferase inhibitors, against 65 clinical isolates of *Candida *spp. by the CLSI reference broth microdilution method.

Drugs	Species (no. of isolates)	Concentration (μg.ml^-1^)		
		
		range of the MICs	^+^MIC_50_	^+^MIC_90_
AZA	All species (65)	≤ 0.03 – > 16	0.5	2
	*Candida albicans *(21)	0.06 – > 16	0.5	8
	*Candida parapsilosis *(19)	0.06 – > 16	0.12	0.5
	*Candida tropicalis *(14)	0.06 – > 16	0.62	8
	*Candida glabrata *(2)	0.12 – > 16	1	2
	*Candida krusei *(1)	16 – > 16	16	> 16
	*Candida lusitaneae *(1)	0.06 – 0.5	0.06	0.5
	*Candida guilliermondii *(3)	≤ 0.03 – 0.5	0.06	0.25
	*Candida zeylanoides *(1)	≤ 0.03	≤ 0.03	≤ 0.03
	*Candida rugosa *(1)	0.25 – 1	0.25	1
	*Candida dubliniensis *(1)	0.5 – 2	0.5	2
	*Candida lipolytica *(1)	> 16	> 16	> 16
				
EIL	All species (65)	≤ 0.03 – > 16	2	2
	*Candida albicans *(21)	0.5 – 8	2	2
	*Candida parapsilosis *(19)	0.5 – 8	1	2
	*Candida tropicalis *(14)	1 – 8	1	2
	*Candida glabrata *(2)	0.5 – 4	1	2
	*Candida krusei *(1)	8	8	8
	*Candida lusitaneae *(1)	0.5 – 2	0.5	2
	*Candida guilliermondii *(3)	1 – 4	1	4
	*Candida zeylanoides *(1)	1 – 2	1	2
	*Candida rugosa *(1)	1 – 2	1	2
	*Candida dubliniensis *(1)	2 – 8	2	8
	*Candida lipolytica *(1)	≤ 0.03	≤ 0.03	≤ 0.03

^+^MIC results are medians.

### Correlations between MIC values

Positive correlations of the MIC_50 _values were observed between AZA and AMB (r = 0.47), AZA and EIL (r = 0.46), and FLC and ITC (r = 0.79). In addition, positive correlations were observed between the MIC_90 _values of the FLC and ITC (r = 0.71). On the other hand, no significant correlations were observed between the MIC values for azoles and 24-SMTI. Some clinical isolates with a trailing effect for FLC (n = 17) and ITC (n = 11) also showed residual growth at higher concentrations of AZA (16 μg.ml^-1^) of 58% (10/17) and 54% (6/11) of the isolates, respectively. Residual growth was not observed in the isolates after treatment with EIL.

### Minimum fungicidal concentration (MFC) of AZA and EIL

The MFCs obtained for half of our isolates were higher than 16 μg.ml^-1^, revealing a predominant fungistatic activity of the SMTI. Interestingly, 4 CNA isolates (*C. glabrata, C. lusitaneae*, *C. zeylanoides*, and *C. rugosa*) showed MFCs lower than 4 μg.ml^-1^, indicating a remarkable fungicidal activity, especially for AZA (Table 4). On the other hand, AZA killed a negligible number of the *C. albicans *isolates at concentrations lower than 16 μg.ml^-1^.

**Table 4:** Cumulative MFC profile of 65 clinical isolates of *Candida *spp. treated with 20-piperidin-2-yl-5α-pregnan-3β,20-diol (AZA) and 24(R,S),25-epiminolanosterol (EIL).

		Cumulative MFC* (μg.ml^-1^)						
Species (no. isolates)	Drugs	0.03	1	2	4	8	16	> 16

All species (65)	AZA	1.52	3.04	12.16	16.72	34.96	44.08	100
	EIL			6.08	15.20	30.40	51.68	100
*Candida albicans *(21)	AZA			4.76	4.76	9.52	9.52	100
	EIL				9.52	28.57	61.98	100
*Candida parapsilosis *(19)	AZA		5.26	26.31	36.87	68.42	68.42	100
	EIL			10.52	15.79	26.31	63.15	100
*Candida tropicalis *(14)	AZA					35.71	64.28	100
	EIL			7.17	7.17	35.71	42.87	100
*Candida glabrata *(2)	AZA			50	50	50	50	100
	EIL				50	50	50	100
*Candida krusei *(1)	AZA							100
	EIL							100
*Candida lusitaneae *(1)	AZA							100
	EIL				100	100	100	100
*Candida guilliermondii *(3)	AZA							100
	EIL							100
*Candida zeylanoides *(1)	AZA	100	100	100	100	100	100	100
	EIL			100	100	100	100	100
*Candida rugosa *(1)	AZA				100	100	100	100
	EIL							100

* data is expressed in percentual of isolates.

### Ultrastructural effects

**Figure 2:**
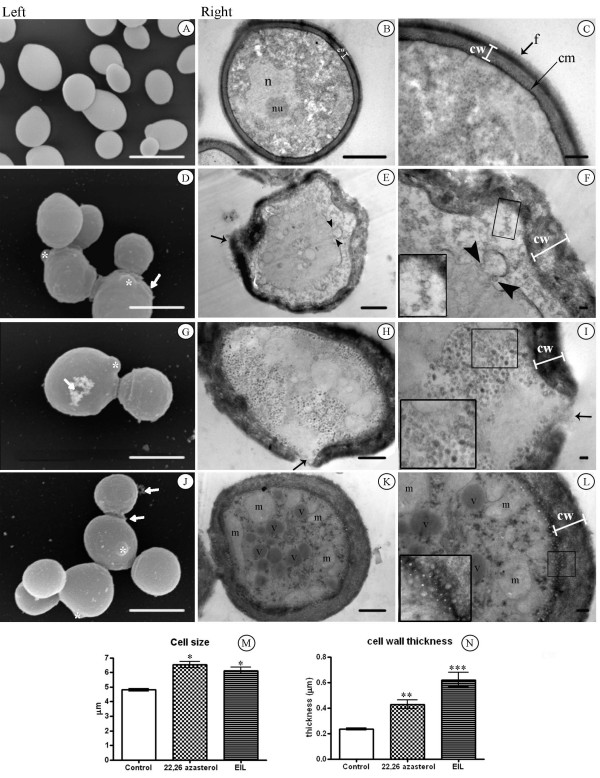
**Scanning electron microscopy (left column) and transmission electron microscopy (two right columns) micrographs of *C. albicans *(isolate 77) untreated (Fig. A-C) and treated with MIC_50 _of AZA (0.25 μg.ml^-1^) (Fig. D-F) and EIL (1 μg.ml^-1^) (Fig. G-L) for 48 h at 35°C**. Control cells have a normal ultrastructure, with nucleus (n), nucleoli (nu), continuous cytoplasmatic membrane (cm), compact cell wall (cw) with fibrillar structures (f), and several ribosomes in the cytoplasm (Fig. A-C). Treated cells show ultrastructural alterations, such as: presence of small buds (asterisks in Fig. 2D, G and J); cell-wall disruption (black and white arrows in Fig. D-J), and increased thickness (cw in Fig. F, I and L); budding of small vesicles coming from the intracellular membranes (arrowhead in Fig. F); accumulation of small vesicles in the periplasmatic region (inset in Fig. F), in cytoplasm (inset in Fig. I), and in close association with the cytoplasmatic membrane (inset in Fig. L); accumulation of electron-dense vacuoles (v in Fig. K) and mitochondrial swelling (m in Fig. K). The effect of 24-SMT inhibitors on cell size and on cell wall thickness was measured and statistically analysed (Fig. M and N, respectively). Bars in A, D, G, and J = 5 μm; B, E, H, and K = 1 μm; C, F, I, and L = 0.2 μm. * p < 0.01; **p < 0.001; ***p < 0.0001.

The general morphology of untreated *C. albicans *was observed using scanning (Figure 2a) and transmission (Figure 2b–c) electron microscopy. The shape of *C. albicans *varies from spherical (4.90 ± 0.49 μm diameter) to oval cells when viewed by scanning electron microscopy (Figure 2a). Transmission electron microscopy revealed the presence of normal cell walls with a thickness of 233 ± 25 nm (Figure 2b–c), including a thin electron-dense outer layer with delicate fibrillar structures clearly visible (f in Figure 2c). A continuous cytoplasmatic membrane (cm)lining a homogeneous and electron-dense cytoplasm containing ribosomes, nucleus (n), and nucleoli (nu) could also be observed (Figure 2b–c). Treatment of *C. albicans *with MIC_50 _of AZA (0.25 μg.ml^-1^) and EIL (1.00 μg.ml^-1^) induced significant morphological changes, which ranged from discrete alterations to total destruction of the fungal cells. A common alteration observed after the treatment with AZA and EIL was a significant increase in cell size, from 5 μm to 7 μm in diameter (Figure 2d, g, j, and 2m). The number of altered cells was counted, and the morphological alterations appeared in 34.79% and 55.17% of the cells after treatment with AZA and EIL, respectively. Among the most frequently observed ultrastructural alterations were: (i) presence of small buds (asterisks in Figure 2d, g and 2j); (ii) irregular cell-wall surfaces (arrows in Fig. 2D and 2E); (iii) loss of cell-wall integrity, with an apparent shedding of cell components (Fig. 2G–J, white and black arrows); and (iv) a two- to three-fold increment of the cell wall thickness was observed after treatment with AZA and EIL, respectively (Figure 2f, i, l, and 2n). The cytoplasmic membrane of treated cells also showed several changes, such as the presence of evaginations, discontinuity, detachment from the cell wall (Figure 2e–f); budding of small vesicles that could migrate through the periplasmatic region (Figure 2e–f, black arrowhead), accumulate in the cytoplasm (Figure 2h–i, details in box), or remain close to the cell membrane (Figure 2k–l, details in box). Vesicle sizes ranged from 40–80 nm for AZA and 40–220 nm for EIL. Mitochondrial swelling and electron dense vacuoles accumulation was also observed (m, Figure 2k–l). CNA cells treated with MIC_50 _of 24-SMTI showed similar ultrastructural changes (data not shown).

### Presence of lipid bodies

**Figure 3:**
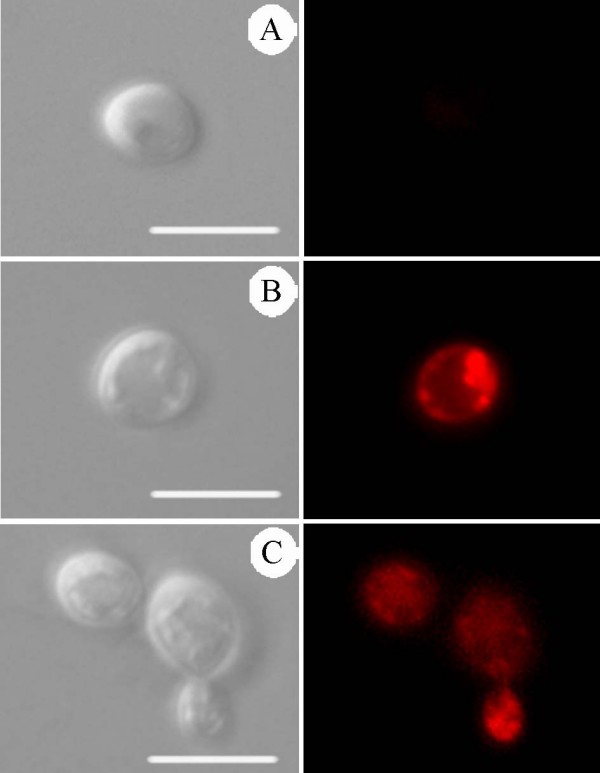
**Differential Interference Contrast (DIC) microscopy (left) and fluorescence microscopy with Nile Red (right) of *C. albicans *(isolate 77) control (A), treated with MIC_50 _of AZA (0.25 μg.ml^-1^) (B) and EIL (1 μg.ml^-1^) (C) for 48 h at 35°C, showing the presence of lipid droplets**. Bars = 5 μm.

Treatment with MIC_50 _of AZA and EIL induced an accumulation of lipid bodies in the cytoplasm, which can be characterised by the presence of small dots labelled with Nile Red (Figure 3b–c), which were absent in the untreated yeasts (Figure 3a). These lipid bodies seen by fluorescence microscopy can be correlated with the small, electron-dense vacuoles seen by transmission electron microscopy (see above, ultrastructural effects).

### Effect of 24-SMT inhibitors on the cell cycle

**Figure 4:**
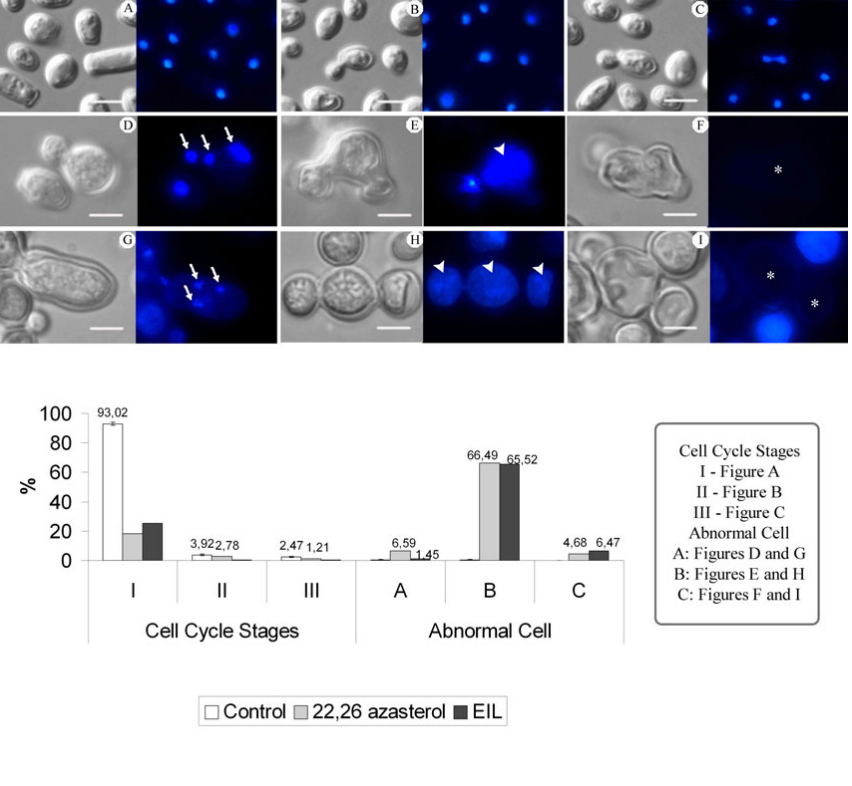
**Differential Interference Contrast (DIC) microscopy (left) and fluorescence microscopy with DAPI (right) of *C. albicans *(isolate 77) control and treated with MIC_50 _of AZA and EIL, showing alterations in the cell cycle such as the presence of cells with multiple nuclei (arrows in Fig. D and G), abnormal chromatin condensation (arrowheads in Fig. E and H), and cells without a nucleus (asterisk in Fig. F and I)**. **A-C: **control cells in different stages of the cell cycle; **D-F: **0.25 μg.ml^-1 ^AZA; **G-I: **1 μg.ml^-1 ^EIL; **J: **Percentage of *C. albicans *cells, untreated and treated with 24-SMT inhibitors, showing different cell cycle stages: (I) cells with no bud and one nucleus, (II) cells with a bud and one nucleus, and (III) cells with a bud and two nuclei (one in each cell); and alterations of cell cycles: (A) cells with more than one nucleus, (B) cells showing abnormal chromatin condensation, and (C) cells without a nucleus. Bar = 5 μm.

DAPI staining used to label the DNA revealed that the treatment of *C. albicans *with AZA and EIL induced important alterations in the cell cycle (Figure 4). To quantitatively assess these alterations, different stages of the untreated yeasts were considered, such as: (I) cells containing one nucleus, (II) cells with a bud and one nucleus, and (III) cells with a bud and two nuclei (one in each cell). After treatment with the MIC_50_s of AZA and EIL, different alterations in the nucleus were observed, and these were classified as: (A) cells with more than one nucleus, (B) cells showing abnormal chromatin condensation, and (C) cells without a nucleus. Counting the number of abnormal cells revealed that approximately 66% of the yeasts showed abnormal chromatin condensation, whereas 6.6% of AZA-treated and 1.5% of EIL-treated cells contained more than one nucleus, and approximately 6% of the cells treated with both compounds had no nucleus (Figure 4).

### Cytotoxicity evaluation

Cytotoxicity of 24-SMTI was evaluated against mammalian cells (Vero) using the sulforhodamine B viability assay. For both AZA and EIL the CC_50 _was 40 μg.ml^-1^, which corresponds to a mean selectivity index of 80 for AZA and 20 for EIL.

## Discussion

Although *C. albicans *is the predominant species in candidiasis, CNA species have increased in frequency in recent years. The reasons for the emergence of CNA species are not fully understood, but some medical conditions may frequently run the risk of developing candidaemia due to the CNA species: *C. parapsilopsis *has been associated with vascular catheters and parenteral nutrition; *C. tropicalis *with cancer and neutropenia; and *C. krusei *and *C. glabrata *with previous treatments with FLC and ITC [2].

Previous studies have described a high susceptibility of *C. albicans *isolates to azoles and AMB, whereas CNA isolates are usually less susceptible and may be intrinsically resistant to FLC and ITC [2,15-17]. As reported by other investigators [2,18,19], none of our *Candida *isolates showed MIC ≥ 2 μg.ml^-1 ^for AMB. MIC values found for ITC and FLU were similar to those previously reported by different groups [2,15-17]. However, in the present study, FLC-resistant *Candida *strains were only observed among CNA species (6.8% of the isolates). However, ITC-resistance was found in *C. albicans *(1.5%) and CNA isolates (7.7%).

The observation of a high positive correlation between MIC values of FLC and ITC (r = 0.79 for MIC_50 _and r = 0.71 for MIC_90_), in this study, suggests that cross-resistance may be occurring. However, no correlation was observed between MIC values of the azoles and 24-SMTI, indicating lack of possible cross-resistance.

The general finding for our *Candida *spp. isolates was that they were mostly susceptible to AZA and EIL, because the MIC_50_s were lower than 2 μg.ml^-1 ^for 73% and 88% of the isolates after treatment with AZA and EIL, respectively. Interestingly, some FLC- and ITC-resistant strains were susceptible to 24-SMTI. However, residual growth of *Candida *after treatment with AZA was similar to that observed for FLC and ITC. No residual growth was observed after treatment with EIL. The fungicidal action of 24-SMTI was more prominent against CNA species than against *C. albicans *isolates. A concentration of 4.0 μg.ml^-1 ^of 24-SMTI was enough to kill 100% of *C. lusitanae*, *C. zeylanoides*, and *C. rugosa*, and 50% of *C. glabrata*. In contrast, this same concentration killed only 4.7% and 9.5% of *C. albicans *isolates, considering AZA and EIL respectively.

Previous studies have shown that azasterol derivatives have antifungal activity against a variety of species [7]. 15-azasterol, in concentrations ranging from 0.01 μg.ml^-1 ^to 4.08 μg.ml^-1^, inhibits the growth of *Saccharomyces cerevisae *and *C. albicans*, with a concomitant accumulation of sterol intermediate molecules [20,21]. The range of MIC and MFC values for 15-azasterol analogues against these fungal species varied from 0.8 to 3.1 μg.ml^-1 ^and 3.1 to 6.3 μg.ml^-1^, respectively [7] and are similar to the values obtained in the present study.

Other azasterol derivatives have been shown to inhibit *S. cerevisae *24-SMT, leading to the accumulation of zymosterol [22]. Recent work demonstrated that AZA displays antifungal activity against *Paracoccidioides brasiliensis *[14] and *Pneumocystis carinii *[13]. Concentrations of 5 μM (2.05 μg.ml^-1^) inhibited 100% of the growth in *P. brasiliensis*, and the treatment of *P. carinii *with the IC_50 _of 0.3 μM (0.12 μg.ml^-1^) led to growth arrest and accumulation of 24-desakyl sterols, indicating an inhibition of 24-SMT [13]. In addition, previous studies have also shown an anti-protozoan activity of AZA and EIL on *T. cruzi *epimastigotes and intracellular amastigotes [10], *L. amazonensis *promastigotes and intracellular amastigotes [11,12], *Toxoplasma gondii *[23], and *Giardia lamblia *[24], with MICs in the low μM to sub-μM range. For protozoans, EIL was reported to be more active than AZA. In contrast, we found in this study that AZA was more active than EIL against *Candida *spp. isolates.

Treatment of *C. albicans *yeasts with AZA and EIL caused dramatic changes in their cellular and sub-cellular structure. The main alterations included changes in the cell wall shape and thickness, a pronounced disconnection between the cell wall and cytoplasm, with the presence of an electron-lucent zone between them, cell collapse and release of cellular content, mitochondrial swelling, and abnormalities in the nuclear structure. These findings are similar to those previously reported after treatment of *Candida *spp. with different azoles [25-28]. Borges and co-workers [27] reported that exposure of *Candida albicans *to ITC leads to primary alterations at the cell periphery and the appearance of vacuoles in the cytoplasm, which may be lipid inclusions. These changes were usually accompanied by an increase in the cell volume and impaired cell division. In addition, studies by Hazen and co-workers [28] revealed that *Candida *treated with FLC shows a distinct retraction of the membrane from the cell wall. On the other hand, *C. albicans *treated with low concentrations of AMB shows chromatin condensation and margination, separation of the nuclear envelope, and nuclear fragmentation [29]. High concentrations of AMB induce cellular changes characteristic of necrosis, showing many large vacuoles [29]. Additionally, Bahmed and co-workers [30] demonstrated an increase in cell wall thickness of *Candida *yeasts, which may be related to alterations in the cell wall composition induced by the treatment with AMB.

In addition, similar to our findings, the appearance of multivesicular bodies and myelin-like structures were reported after treatment of *Leishmania *[11,12] and *T. cruzi *[31] with AZA and EIL.

Staining with Nile Red revealed the presence of lipid accumulation in the cytoplasm after treatment with 24-SMTI, confirming that these compounds induce a perturbation in lipid biosynthesis. Similar observations have recently been made as the result of treatment of *Leishmania amazonensis *with 24-SMTI, which induced several abnormalities in the lipid content, with the accumulation of steroid intermediate molecules [12].

In addition, staining of DNA with DAPI indicates a profound alteration in the cell cycle after treatment with AZA and EIL. *Candida *yeasts produced unfertile buds that remained closely associated with the mother cell, and appeared with or without various nuclei. The nucleus may also have an altered shape and/or with abnormal chromatin condensation that might be associated with apoptosis cell death, as previously described after treatment of *C. albicans *with AMB [29]; and also after treatment of *Tritrichomonas foetus *with hydrogen peroxide [32]. The presence of many cells with more than one nucleus may also indicate that ergosterol biosynthesis inhibitors are interfering with cytokinesis. In fact, it was previously found that ergosterol levels modulate the activity of protein kinases such as pp60v-src and also the levels of cAMP, both of which are directly related to the control of the cell cycle [33,34].

In addition, some studies have shown that drugs such as griseofulvin and nocodazole, which interfere with the assembly of cytoskeleton components, induce alterations in the cell cycle and apoptosis cell death [35-37]. Thus, there are two possible explanations for the alterations in the cell cycle: (a) the depletion of ergosterol and other lipids, which are essential for the maintenance of the cytoplasmatic membrane structure and are also important key regulators of the cell cycle; and/or (b) alterations in the cytoskeleton structure, which can affect the cytokinesis process. The results obtained here suggest that AZA and EIL are probably interfering with sterol biosynthesis in *Candida *spp., as previously described for *C. albicans *[20], *P. carinii *[13], *T. cruzi *[3], and *L. amazonensis *[12]. On the other hand, we cannot exclude the possibility that these compounds may be acting in other pathways, inducing some secondary effects that could be related to the accumulation of other lipids or, as demonstrated in *Crithidia deanei*, that AZA can interfere with phospholipid biosynthesis [38]. Further studies are necessary to characterise the correlation between the depletion of ergosterol and the cell cycle in *C. albicans*.

## Conclusion

The results presented herein demonstrate the potential usefulness of the 24-SMT inhibitors AZA and EIL as antifungal agents, including azole-resistant *Candida *strains. The specific *in vitro *and *in vivo *antifungal and antiprotozoal activity of azasterols has been known for years, and in most cases has been linked to their specific inhibition of 24-SMT, an enzyme absent in mammals [10-14,39]. However, other studies have found that these compounds are also active against parasitic protozoa that lack endogenous sterol biosynthesis, such as *T. gondii *[23,40] and *Trypanosoma brucei *[41], indicating that they may have other biochemical targets. Taken together, these results indicate azasterols as useful leads for novel antifungal agents, but optimisation of their selectivity, ADME, PK, and toxicological properties is required for their further advancement as drug candidates.

## Methods

### Microorganisms

Antifungal assays were performed against 70 yeasts of the genus *Candida*. Five standard strains from the American Type Culture Collection (ATCC): *Candida albicans *ATCC 10231, *Candida krusei *ATCC 6258, *Candida glabrata *ATCC 2001, *Candida parapsilosis *ATCC 22019, and *Candida tropicalis *ATCC 13803; and 65 clinical isolates: *Candida albicans *(21), *Candida parapsilosis *(19), *Candida tropicalis *(14), *Candida guilliermondii *(3), *Candida glabrata *(2), *Candida krusei *(1), *Candida lusitaneae *(1),*Candida zeylanoides *(1), *Candida rugosa *(1),*Candida dubliniensis *(1), and *Candida lipolytica *(1) were used. The clinical isolates came from bloodstream (35%), urine (26%), and other clinical material (39%), and were isolated from 2002 to 2006 at the Microbiology/Mycology Laboratory of Hemorio, Rio de Janeiro, Brazil. Species identification was performed by micromorphology analysis and Vitek Systems (Biomerieux Inc., France). The isolates were maintained in Sabouraud dextrose agar plates at 4°C, and subcultures were used in each experiment.

### Drugs

20-piperidin-2-yl-5α-pregnan-3β, 20-diol (22,26-azasterol or AZA) (Fig. 1) and 24(R,S),25-epiminolanosterol (EIL) (Fig. 1), Δ^24(25)^-sterol methyltransferase inhibitors, were synthesised, purified, and characterised as described by Urbina et al. [10]. Fluconazole (FLC) (Pfizer, São Paulo, Brazil), Itraconazole (ITC), and Amphotericin B (AMB) (both from Sigma Chemical Co., Missouri, USA) were used as reference antifungals. Drugs were diluted in dimethyl sulfoxide (DMSO) to obtain 100-times stock solutions and maintained at -70°C.

### Antifungal susceptibility test

The minimal inhibitory concentration (MIC) of each drug was obtained using the broth microdilution technique as described in document M27-A3 of the Clinical and Laboratory Standards Institute – CLSI [42]. Briefly, serial two-fold dilutions of the drugs were performed in RPMI 1640 medium (Sigma Chemical Co., Missouri, USA), buffered with MOPS 0.16 M, pH 7.0, into 96-well microtitre trays to obtain concentration ranges of 0.03–16 μg.ml^-1 ^(AZA, EIL, and ITC), 0.25–128 μg.ml^-1 ^(FLC) and 0.007–4 μg.ml^-1 ^(AMB). Next, the yeast inoculum was adjusted to 1–5 × 10^6^CFU.ml^-1^. Dilutions of 1:50 and 1:20 in RPMI 1640 medium were performed to obtain 1–5 × 10^3 ^CFU.ml^-1^, and an aliquot was dispensed into each well. The microtitre trays were incubated at 35°C, for 48 h. MIC_50 _and MIC_90 _values (MICs that inhibit 50% and 90% of the yeast growth in relating to control, respectively) were determined using a spectrophotometer at 492 nm. MIC_50 _and MIC_90 _median values for test and standard drugs were also determined. Clinical isolates were classified according to their MIC in three different categories: susceptible (S), susceptible dose-dependent (SDD), or resistant (R). Interpretative breakpoints proposed by the CLSI [42] for FLC and ITC were used, and concentrations above 1 μg.ml^-1 ^were considered resistant for AMB [43]. Trailing effect for FLC and ITC was detected at visual reading after 24 h of incubation.

The minimum fungicidal concentration (MFC) was determined after 48 h of treatment with the inhibitory concentrations used in the susceptibility test. An aliquot of each *Candida *isolate was transferred onto Sabouraud dextrose agar plates without the presence of drugs. The plates were incubated at 35°C for 48 h, and the minimum fungicidal concentration (MFC) was determined. MFC means the lowest concentration that showed no fungal growth [44].

### Fluorescence microscopy

*C. albicans *(isolate 77) was treated with MIC_50 _of AZA and EIL at 35°C for 48 h. Yeasts were washed in PBS, pH 7.2 and fixed with 4% paraformaldehyde in PBS for 30 min. Next, the yeasts were adhered to coverslips with poly-L-lysine and incubated with 5 μg.ml^-1 ^Nile Red (Fluka, USA) for 30 min to label the lipid bodies and 1 μg.ml^-1 ^DAPI (Sigma Chemical Co., Missouri, USA) for 10 min to label the DNA. Coverslips were mounted in n-propylgallate solution and observed in a Zeiss Axioplan epifluorescence microscope equipped with rhodamine (Nile Red fluorescence) and DAPI filters, and the images were recorded with a C5810 Hamamatsu camera. The number of altered *Candida *was determined after the counting of at least 300 yeast cells. Cell size was measured by means of the SemAfore 5.0 software (Jeol, Japan).

### Transmission electron microscopy

*C. albicans *(isolate 77) was treated with MIC_50 _of AZA and EIL at 35°C, for 48 h. Yeasts were washed in PBS, pH 7.2 and fixed in a solution of 2.5% glutaraldehyde and 4% freshly prepared formaldehyde in 0.1 M cacodylate buffer, pH 7.2, for 2 h at room temperature. After fixation, yeasts were post-fixed for 2 h in 1% osmium tetroxide containing 1.25% potassium ferrocyanide and 5 mM CaCl_2 _in cacodylate buffer, pH 7.2, washed in the same buffer, dehydrated in ethanol, and embedded in Spurr. Ultrathin sections were stained with uranyl acetate and lead citrate, and images were obtained in a Zeiss 900 electron microscope equipped with a CCD Camera (Mega view III model, Soft Image System, Germany). Images were processed with iTEM software (Soft Image System, Germany). Cell wall thicknesses and vesicles of untreated and treated yeasts were measured by means of the SemAfore 5.0 software (Jeol, Japan).

### Scanning electron microscopy

*C. albicans *(isolate 77) treated with MIC_50 _of AZA and EIL at 35°C for 48 h, was fixed as described above for transmission electron microscopy, and subsequently dehydrated in ethanol, critical-point dried in CO_2_, coated with gold, and observed in a JEOL JSM-5310 scanning electron microscope.

### Cytotoxicity tests in mammalian cells

Green monkey kidney (Vero) cells were maintained in Dulbecco's modified Eagle's medium (DMEM, Gibco Invitrogen Corporation, New York, USA) supplemented with 2 mM L-glutamine, 10% heat-inactivated fetal bovine serum (FBS), and 50 μg.ml^-1 ^gentamicin at 37°C in a 5% CO_2_/air mixture. In 96-well microtitre trays, 2.5 × 10^4 ^cells/well were dispensed and incubated for 24 h. Monolayers of Vero cells were treated with different concentrations of 24-SMTI for 48 h at 37°C in 5% CO_2 _and fixed in 10% trichloroacetic acid for 1 h at 4°C, stained with sulforhodamine B for 30 min at 4°C, and the optical densities were obtained in a spectrophotometer at 530 nm [45]. The 50% cytotoxic concentration (CC_50_) and the selectivity index (SI = CC_50_/MIC_50_) were calculated.

### Statistical analysis

Statistical analyses were performed with the Prism 5.0 software, and p < 0.05 was considered as significant. Differences in the cell size and cell-wall thickness of untreated and treated *Candida *spp. were analysed by one-way ANOVA (Dunnett test), and MIC values were analysed by linear regression test.

## Authors' contributions

KI, JCFR and SR designed the study and wrote the manuscript. The syntheses of 24-SMT inhibitors were performed by JAU. MDR provided the clinical isolates. KI and TVMV realized the susceptibility assay, fluorescence and transmission electron microscopy. CVN worked on cytotoxicity tests. JAU and WS critically revised the manuscript for its important intellectual content. All authors read and approved the final manuscript.
